# Propagation and Introduction of *Arnica montana* L. into Cultivation: A Step to Reduce the Pressure on Endangered and High-Valued Medicinal Plant Species

**DOI:** 10.1155/2013/414363

**Published:** 2013-10-24

**Authors:** Danuta Sugier, Piotr Sugier, Urszula Gawlik-Dziki

**Affiliations:** ^1^Department of Industrial and Medicinal Plants, University of Life Sciences in Lublin, Akademicka 15, 20-950 Lublin, Poland; ^2^Department of Ecology, Faculty of Biology and Biotechnology, Maria Curie-Skłodowska University, Akademicka 19, 20-033 Lublin, Poland; ^3^Department of Biochemistry and Food Chemistry, University of Life Sciences, Skromna 8, 20-704 Lublin, Poland

## Abstract

*Arnica montana* (L.) is an endangered and endemic medicinal plant species in Europe. The pressure on natural sources of this plant is alleviated by a suitable use of arnica resources in the European region and introduction into cultivation. The objective of this study was to describe the impact of different ways of plant propagation and introduction on the growth and reproduction mode of this species. During the six consecutive years of the field experiment, the vegetative and reproductive traits were monitored, and survival time was assessed. The particular ways of arnica plant propagation and introduction determined all the intrinsic species traits and plant survival. The values of the characteristics studied indicated good acclimatization of the arnica ecotype to the climatic conditions of eastern Poland. Practical implications from the data presented here include the possibility of using the presented modes of arnica propagation and introduction in the short- and long-term perspective of arnica cultivation, which can give a possibility of better adjustment of raw material production.

## 1. Introduction 

*Arnica montana* L. (Asteraceae) is a rhizomatous herbaceous perennial herb and a medicinal plant widely used as a herbal remedy occurring throughout the entire range of the species, from South Norway and Latvia southwards to the Apennines and the South Carpathians [1]. This species prefers acid and poor soil conditions. It is mainly found in grasslands and shrublands and alpine mountain environments. *A. montana* also grows in dry pine forests, meadows on siliceous soils, marginal parts of spruce forests, open forest edges, mowing pastures, and margins of peatlands [2–7]. The species is a component of the five habitat types of the Habitats Directive [8].

Eutrophication, habitat fragmentation, and agricultural intensification have led to a rapid decline in the mountain arnica in many European countries [9, 10]. *A. montana* habitats have been fragmented, especially at the edge of its dense geographical range [11]. The negative influence on the arnica population comes from such competitors as *Dactylis glomerata* [12], *Agrostis capillaris* [7], *Deschampsia flexuosa*, and *Calamagrostis villosa *[10]. Additionally, the main serious damage is caused by slug herbivores and the specialist fly *Tephritis arnicae*—highly specialized on *A. montana,* the only parasite found in mountain arnica flower heads being a limiting factor for the geographical and mountain altitude range of this species [13, 14].

*Arnica montana* is a medicinally important plant species widely applied in pharmacy, homeopathy, and cosmetics. Different plant parts such as inflorescences, rhizomes, roots, and leaves are collected for healing purposes. Arnica is a source of sesquiterpenes, essential oils, terpenoids, sesquiterpene lactones, flavonoids, and phenolic acids, especially chlorogenic acids [15–17] and exhibits antiseptic, anti-inflammatory, antibacterial, antisclerotic, antifungal, and antioxidant activities [15, 18]. Recently, new derivatives of chlorogenic acids in *A. montana* flowers and the composition of essential oils in rhizomes and roots have been identified and characterized [19, 20].

*A. montana* is a rare plant under strict protection and is included in the IUCN Red List of Threatened Species [8] and in the Red Data Books and Red Data Lists of many European countries [21, 22]. Despite the loss of habitats, *A. montana *is mainly harvested from the wild. Dried flowers traded annually in Europe are estimated to be around 50 t [23]. The collection of *A. montana* for medicinal purposes has also caused disappearance or reduction in the size of several European populations [24]. The pressure on natural sources of this plant is alleviated by a suitable use of arnica supply in the European region, where flower heads are harvested [25]. *A. montana* is protected by law; however, this form of protection is insufficient. Therefore, over the last decades, other forms of active protection have appeared such as restoration and management of its natural habitats [9, 26, 27], plant introduction [27, 28], and attempts at field cultivation [20, 29, 30]. Biotechnological approaches for cultivation and enhancement of secondary metabolites in these species are very interesting [31, 32].

The objective of this study was to describe the impact of different ways of plant propagation and introduction on the growth and reproduction mode of this species in the area of its geographical range in the eastern part of Poland and to obtain information on their suitability for climatic and edaphic conditions. During the six consecutive years of the experiment, the vegetative and reproductive traits were monitored as potentially important factors influencing the plant establishment success [33]. In the future, the knowledge of the part of the life history of these species can be helpful in cultivation, which may reduce the pressure on natural population and reintroduction of *A. montana* into their natural stands, where the abundance of arnica has declined.

## 2. Materials and Methods

### 2.1. Experimental Site Conditions

In the present study, we used individuals taken from Botanical Garden UMCS in Lublin. The 6-year-long experiments (2007–2012) were performed on experimental fields at the University of Life Sciences in Lublin located in the eastern part of Poland, 51°33′ N; 22°44′ E on grey-brown podsolic soil with the granulometric composition of heavy loamy sand. This site was chosen because the soil has physicochemical properties comparable to natural soil conditions for arnica [7, 9].

**Figure 1:**
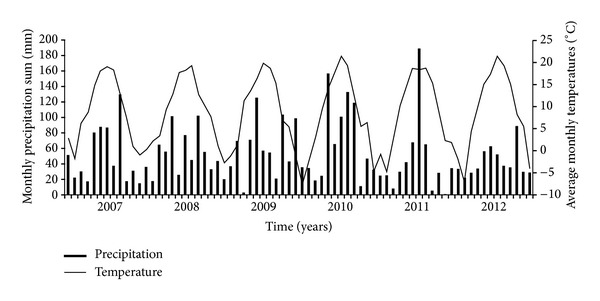
Monthly average temperature and monthly precipitation sum at studied region for six successive study years 2007–2012.

The climate of the Lubelszczyzna region is mainly affected by penetrating polar-oceanic masses of air and polar masses of continental air. The growing season in the middle and eastern part of these region lasts 211–214 days [34]. The average month precipitation ranged from 1.0 mm in November 2011 to 189 mm in July 2011, and the average month temperature ranged from −8.1°C in January 2010 to 21.6°C in the cultivation years 2007–2012 (Figure 1). However, the mean annual sum of precipitation in the study period ranged from 510.2 mm in 2012 to 778.8 mm in 2010, and the mean annual temperature ranged from 7.98°C in 2010 to 9.59°C in 2008.

In order to determine the edaphic conditions before plantation establishment, topsoil samples were randomly selected from the depth of 15 cm. The main soil properties were analysed. The topsoil was characterized by an average content of organic matter (1.55%), high phosphorus (84.1 mg P*·*kg^−1^, PN-R-04023: 1996), average potassium (72.2 mg K*·*kg^−1^, PN-R-04022: 1996+Az1:2002), very low magnesium (2.4 mg Mg*·*kg^−1^, PN-R-04020: 1994+Az1:2004), and acid reaction (pH in H_2_O—4.18 and in 1 mol*·*dm^−3^ KCl—3.79, PN-ISO 10390: 1997).

### 2.2. Plant Material and Experimental Design

The field experiment was established in April 2007 in three ways of plant establishment. The plants were introduced as clone seedlings (CS) and rosettes (RS) and by sowing (SS).

The randomly chosen ramets taken from edge of 6-year-old genets after their natural disintegration just before planting were transplanted individually into the field. The plants are marked as CS.

The nine-month-old rosettes that were established after spontaneous seed germination and originated from the collection; subsequently, they were individually transplanted into small pits. The plants are marked as RS. In another field experiment conducted in recent years to assess the mortality rates of plants, spontaneous seedling recruitment was observed, hence the idea of using thereof for introduction into cultivation.

*A. montana* seeds (achenes) for sowing were collected from 15 individuals from the arnica collection at the end of June 2006 and stored dry and at room temperature. At the end of April 2007, only dark and firm achenes were selected and sown into rows, where soil taken from mother plantation was scattered. Our earlier experiments indicated that soil taken from plants, compared to commonly used standard substrates, exerted a significant impact on seedling emergence and plant growth. After sowing, the soil was slightly pressed to improve the soil contact with the seeds. Ten weeks after sowing, when the seedlings formed rosettes characterized by 3-4 leafs, individuals were left in rows at 20 cm intervals. The plants are marked as SS.

The *A. montana* seeds were sown and the seedlings were transplanted at a 40 × 20 cm distance, thus ensuring almost the same plant densities (12.5 plants/m^−2^). The experimental unit was made up of 75 plants. The experimental design was a randomized complete block with four replications (plots of 6 m^−2^). During the vegetation period, the plantation was three times weeded by hand. We observed vegetative and reproductive traits and survival of genets from the point of view of species biology; therefore, the flower heads were not harvested. Simultaneously, the same characteristics were studied in the other field experiment after harvesting flower heads. The comparable genets from the experiments with and without head harvest did not differ statistically; hence, the harvest of flower heads did not determine the traits studied.

We present changes in the characteristics studied, calculated per genet containing several ramets formed during the vegetative growth. In the last year of observation, we observed disintegration of genets and formation of clone clusters. Despite this fact, we defined the units as genets, that is, genetic individuals containing some ramets.

### 2.3. Measurements under Field Conditions

Each summer, at the peak of flowering, 30 individuals per each mode of plant establishment were measured when available. Several vegetative and reproductive characters were measured: the total number of ramets per genet (TNR), the number of flowering ramets per genet (NFR), the height of flowering stems per genet (HS), the number of heads per genet (NH), the number of heads per flowering stem of the genet (NHFS), and the head diameter per genet. The total area of heads per genet (AH) was calculated on the basis of the head diameter. On the basis of 6-year observation, the cumulative area of heads per genet (CAH) and the cumulative number of heads per genet (CNH) were determined. The morphological and reproductive parameters were recorded from 30 plants/repetition.

The survival time was assessed by recording survival of individual plants each June from the beginning of the experiment until July 2012. A plant was considered dead when it was not found during subsequent censuses. The presented climatic data were obtained from the Felin climatic station.

### 2.4. Statistical Analysis

Prior to the analysis, all data were tested for normality with the Shapiro-Wilk test. Variance heterogeneity was checked using a Levene*ʼ*s test. Parametric data were tested using one-way analysis of variance (ANOVA) with subsequent Tukey tests (data were normally distributed with homogeneous variances). When the data were not normally distributed or the variance was not homogeneous, the differences between the 3 modes of introduction were tested using Kruskal-Wallis tests with subsequent Mann-Whitney *U*-tests. All results are expressed as means ± SD, and the differences were considered significant *P* < 0.05. All statistical analyses were carried out using the Statistica software programme.

## 3. Results

**Figure 2:**
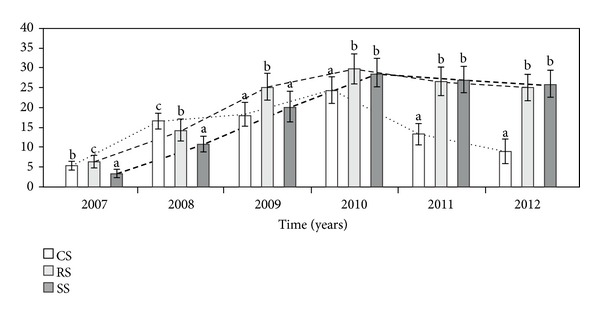
The total number of ramets per genet (TNR) in different ways of *A. montana* introduction into cultivation. The plants were introduced as clone seedlings (CS) and rosettes (RS) and by sowing (SS). Bars (means) followed by the different letters within the same study year differ significantly (Tukey-test, *P* < 0.05).

The total number of ramets per genet (TNR) differed significantly between the three different modes of plant establishment in each study year (Table 1, Figure 2). In the first full vegetation season (2007), plants introduced as CS formed 2–6 new offspring ramets (mean 5.4), and genets introduced as RS formed 3–8 new offspring ramets (mean 6.4). However, individuals grown from seeds produced 2–5 new offspring rosettes (mean 3.4). In the second year of observation, the CS genets had a higher TNR, while in the third year, the RS were characterised by a higher TNR. In the fourth, fifth, and sixth study years, the CS genets had a significant lower TNR per genet than those of the RS and SS; however, there was no significant difference between the RS and SS.

**Figure 3:**
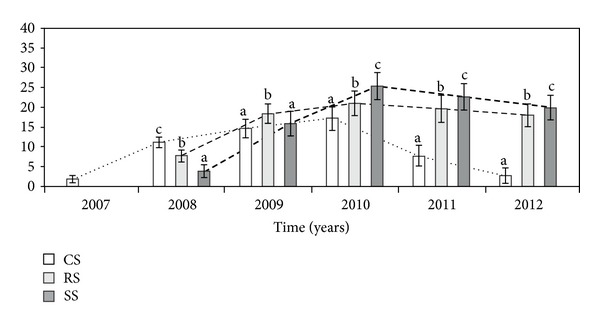
Number of flowering ramets per genet (NFR) in different ways of *A. montana* introduction into cultivation. Explanation: see Figure 2.

In the first year of observation, the genets introduced as RS and SS did not generate flowering stems. However, the mean number of flowering stems produced by individuals grown from clones was 1.8. In each subsequent study year, there was a significant difference in the total number of flowering ramets per genet (NFR) between the three modes of establishment (Table 1, Figure 3). In 2008, the NFRs in the CS were higher than those in the RS and SS. However, in 2009, the NFRS in the RS were higher than those in both the CS and SS genets. In 2010–2012, the number of flowering ramets was significantly higher in the SS plants in comparison to CS and RS. In 2011, the NFR in the CS plants was over two times lower than in both RS and SS, and ca. 7 times lower in 2012, respectively.

**Figure 4:**
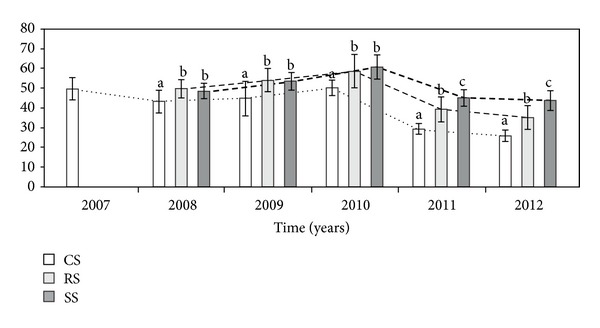
The height (cm) of flowering stems per genet (HS) at different ways of *A. montana* introduction. The plants were introduced as clone seedlings (CS) and rosettes (RS) and by sowing (SS). Bars (means) followed by the different letters within the same of study year differ significantly (Mann-Whitney *U*-test, *P* < 0.05).

In each study year, there was a significant difference in the height of the flowering stems per genet (HS) between the three modes of plant establishment (Table 1, Figure 4). In 2008–2010, the HS in both the RS and SS genets was higher than in the CS plants. In the last two years of observation, the HS in the SS individuals was significantly higher than in the CS and RS genets.

**Figure 5:**
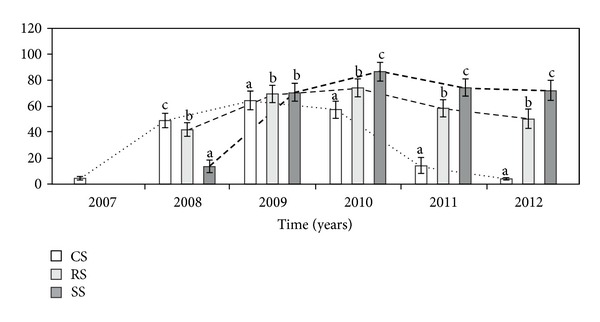
The number of heads per genet (NH) in different ways of *A. montana* introduction. Explanation: see Figure 4.

The number of heads per genet (NH) differed significantly between the three different modes of plant establishment in the all study years (Table 1, Figure 5). In the second vegetation season (2008), plants introduced as CS produced a mean of 49 heads and only in that year their number was significantly higher in relation to genets introduced as RS and SS. In 2009, the NH of both RS and SS genets was significantly higher than in the CS individuals. In the next years of the experiments, the NH in plants introduced as seeds was significantly higher in relation to the RS and CS genets.

**Figure 6:**
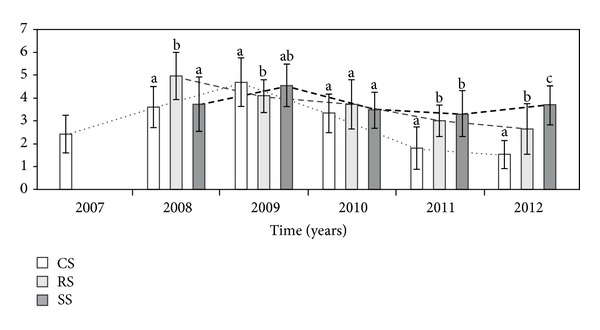
The number of heads per flowering stem of the genet (NHFS) in different ways of *A. montana* introduction. Explanation: see Figure 2.

The number of heads per flowering stem per genet (NHFS) differed significantly between the three different modes of plant establishment, excluding the fourth (2010) year of observation (Table 1, Figure 6). The highest NHFS in the range of 4–8 was produced by RS genets in the second growing season, and the mean value of this parameter was higher than in the CS and SS genets. In the third year of vegetation, the NHFS in the CS genets was comparable to that in the SS plants and significantly higher in relation to the RS genets. However, in the last two years of the experiment, the NHFS in the SS plants was higher than that in the RS and CS genets.

**Figure 7:**
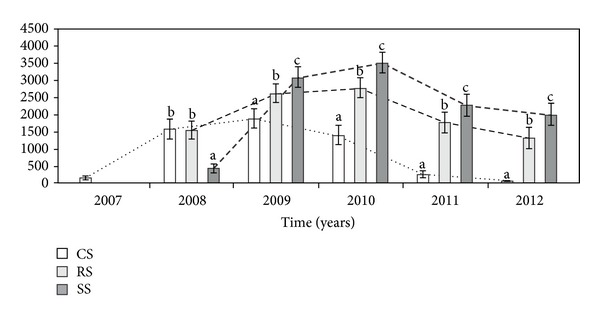
The area of heads (cm^−2^) per genet (AH) in different ways of *A. montana* introduction. Explanation: see Figure 4.

The area of heads per genet (AH) differed significantly between the three different modes of plant establishment in all study years (Table 1, Figure 7). In the second year of observation, the AH of genets introduced as CS and RS was comparable and was over three times higher in relation to plants introduced as seeds. In each consecutive year, the highest area of heads was characteristic for individuals introduced as seeds. In 2009–2012, the AH in both the SS and RS plants was higher than in the CS genets, respectively.

**Figure 8:**
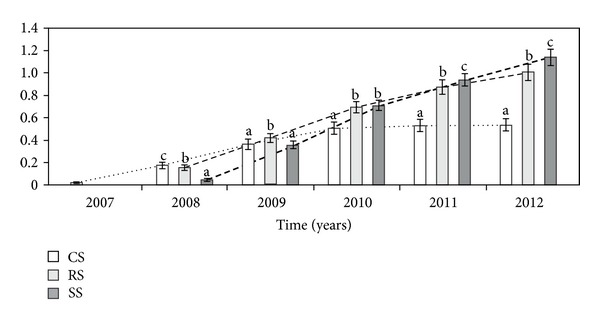
The cumulative area of heads (m^−2^) per genet (CAH) in different ways of *A. montana* introduction. Explanation: see Figure 4.

The cumulative area of heads per genet (CAH) differed significantly between the three different modes of plant establishment in all study years (Table 1, Figure 8). The CAH in genets introduced as CS was higher than that in the case of the other ways of introduction only in the second year of observation (2008). In the third growing season, the CAH in the RS plants was higher than in both the CS and SS genets. In the fourth study year, the CAH in plants introduced as rosettes and by sowing was comparable as well as in relation to the CS plants. However, in the last two years of the experiment, the CAH in plants introduced as seeds was higher than in individuals introduced as rosettes and clone seedlings.

**Figure 9:**
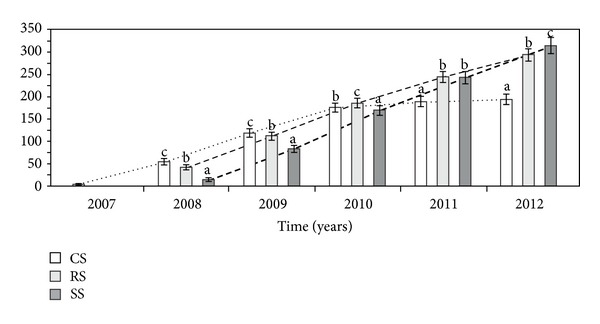
The cumulative number of heads per genet (CNH) in different ways of *A. montana* introduction. Explanation: see Figure 2.

There was a significant difference in the cumulative number of heads per genet (CNH) between the three modes of introduction (Table 1, Figure 9). In the second and third growing seasons, the CNH of genets introduced as CS was higher than in genets introduced as RS and SS, and in the next years of the experiment, it was significantly lower, respectively. In the fourth study year, the CNH was the highest in RS plants, and in the fifth study year, the CNH was comparable in the RS and SS genets. However, in the last year of observation, the CNH in the SS individuals was significantly higher than in the RS and CS plants.

**Figure 10:**
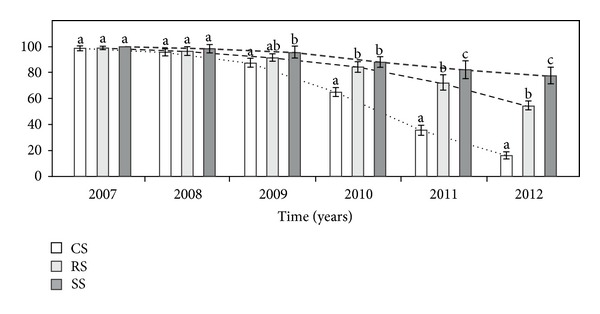
The survival (%) of *A. montana* individuals in different ways of introduction. Explanation: see Figure 4.

In the first two years after plant establishment in the different modes, plant survival was comparable; however, in the consecutive years, the survival differed significantly between the three different modes of plant introduction (Table 1, Figure 10). Nearly six years after introduction, the survival of genets introduced as CS was over 3 times lower than in RS plants and over 4 times lower compared to individuals introduced as seeds.

## 4. Discussion

### 4.1. Propagation and Introduction of *A. montana*

In the different cultivation experiments, the preferable method of establishment for large-scale planting is to raise plants from seeds under glasshouse conditions and transplant them into the field [29, 30, 35, 36]. In our studies, besides sowing in the field, spontaneously established seedlings (rosettes) taken from the arnica collection and edge clones taken from 6-year-old disintegrated genets were used. The ramets and clones were transplanted directly to the ground, without the seedling preparation stage, which can evoke stress in young plants. Simultaneously, it is necessary to underline the reduction of time designed to preparation of seedlings and economical aspect.

One of the ways of plant introduction in the presented studies was using clone seedlings. In studies on arnica introduction on Tara mountain in Serbia, in the variant of spring planting with clonal seedlings taken by dividing 2-year-old genets, the seedlings were almost completely lost [20]. In our studies, we used clonal seedlings taken from 6-year-old clusters, already after genet disintegration; next, the particular ramets were naturally divided and contained parts of rhizomes and roots. Therefore, in the first year of the experiment, the plants formed new ramets and flowered, in contrast to the plants established in the other two modes.

In *A. montana* cultivation, plant survival after sowing or seedling introduction is rarely reported. In an experiment of arnica introduction through seedlings derived from seeds on an abandoned arable field where the top layer had been removed to reduce competition, the survival in the first year after plant establishment was about 50% [28], whereas in our studies, it exceeded 90%. Our data are in good agreement with the results of an experiment on arnica conducted in a natural heath area [27] and are also in agreement with the experiment conducted under controlled greenhouse conditions, where a 90% survival rate was observed when seedlings were inoculated with mycorrhizal fungi [12]. On the contrary, in that experiment, the nonmycorrhizal plants were several times smaller and were dark green, and their survival rate strongly decreased with time, going down to about 16% at the end of the one-year cultivation period.

In the present study, we focused on introduction of plants using seedlings spontaneously established from achenes. During the 6-year study period, rapid growth and reproduction and very low mortality of genets, especially in the first three years of the experiment, were observed. This mode of plant introduction is more likely to eliminate poorly adapted individuals. Moreover, the use of clone seedlings, rosettes, and soil from *A. montana* collection before sowing probably guarantees the presence of mycornhiza, which is always affirmed in arnica natural habitats [37, 38]. This question should be, however, explained and confirmed in further studies.

### 4.2. Vegetative and Reproductive Traits

In other experiments, the vegetative and generative traits of cultivated plants were different and dependent on the mode of plant introduction [20, 30]. This was usually connected with the different age of cultivated plants, especially in the first and second years of plant life. The results of our studies are in agreement with these data and indicate that all the modes of plant establishment determined both the vegetative and reproductive traits. In the case of genets introduced as rosettes and by sowing, the values of the characteristics studied were increased to the fourth year of cultivation and decreased in the next two years. This pattern was in accordance with reports from Finland [35] but is somewhat different from the results of 5-year observation in Serbia [29], where the maximum flower yield was noted in the third year of cultivation.

The number of rosettes per genet reflects clonal growth of plants and is very often counted as a yield component [20]. The values of this parameter in plants introduced by sowing were over 2 times higher compared to data presented from the Alps in plants established in the same way [30]. However, the number of rosettes per genet of 2-year-old and 3-year-old plants was slightly lower in relation to data presented from Tara mountain in Serbia [20]. 

In natural habitats, the proportion of flowering arnica rosettes decreased with altitude, indicating a shift from sexual reproduction to clonal growth and was different in genets which are components of different plant communities [6]. The number of flowering stems per plant was variable and depended mainly on their age. On average, 2-year-old arnica plant established by sowing formed c.a. 5 flowering stems, which was almost 2-fold higher than the results reported by Aiello et al. [30]. In the natural population, not all arnica clusters or rosettes produced generative rosettes and not all individual rosettes within a flowering cluster produced a flowering shoot [2]. Similarly, in the field or glasshouse conditions, plants can produce flowering stems in the next vegetation season after introduction, but the flowering is changeable [13, 28, 30, 39]. However, in the present experiment, the plants introduced as rosettes which were formed in the previous vegetation season did not flower but produced a high number of ramets. The absence of flower heads in genets introduced as seedlings after a cold winter period with a short day, which stimulates flower development [28], can be explained by the fact that *A. montana* is a clonal plant that can allocate resources either to sexual reproduction or clonal growth, that is, production of more rosettes inside genets [6]. In favourable field conditions when plant competition was excluded, arnica genets allocated resources probably in the vegetative growth resulting in a higher number of ramets and lack of flowering at 2-year-old plants.

The total area of flower heads per genet is strictly related to the flower head diameter and reflects the flower head yield [18]. Moreover, the number of seeds per flower head is highly correlated with the flower head diameter and length of the flower stem [4]. In the case of genets established in all the three modes, the value of this parameter was the highest in the fourth year of cultivation. Similarly, the number of flower heads per plant reflects the flower heads yield and in many cases is dependent on the location of the population and site conditions [40]. The values of this parameter in plants introduced by sowing were over 2 times higher compared to the data reported from the Alps in 2-year-old plants established in the same way [30]. The maximum number of flower heads in the fourth year of cultivation did not exceed 100, whereas many highly productive clones selected from wild plant populations were characterized by 134 flower heads per plant [40].

### 4.3. Implication for Cultivation

From the point of view of herb material production, all the presented three ways of introduction can be applied. In the first and second vegetation seasons, arnica plants introduced as ramets taken from 6-year-old plants after genet defragmentation produced the highest number of flower heads and exhibited the highest total area of flower heads. Moreover, the plants generated the highest number of ramets per genet and the greatest number of flowering stems. Therefore, when collection of arnica flowers is planned for 2 or 3 successive years, the plantation should be established by introduction of clone seedlings. This mode of introduction guarantees high crops of *Arnicae Flos* already in the first two years of cultivation.

The number of stems per plant and the number of flowers per plant in 2-year-old genets introduced in this way were over 2 times higher in relation to plants cultivated in Finland [35], introduced as seedlings prepared from seeds and over 50% higher in relation to our earlier studies [18]. The number of flowering stems per plant was over two times higher in relation to plants introduced by sowing in this experiment and almost 2 times higher compared to other results [18, 35]. Therefore, if there is an immediate demand for raw material, the *A. montana* plantation can be established by transplantation of clone seedlings taken from many-year-old genets after their disintegration. However, the very high clonal growth favours production of rhizomes and roots, which are the main source of essential oils [17, 20]. The correlation between the rosette number and rhizome yield is high [20]. In our experiment, plants established by ramets had the highest number of rosettes per genet; however, this way of plant establishment can be used for arnicarhizome material production.

In the longer perspective of arnica cultivation and raw material collection, the two other modes of introduction can be considered. After 3 vegetative seasons, the genets introduced by rosettes from the mother plantation are characterized by the highest cumulative total area of flower heads per genet. However, in the perspective of collection of flower heads in the 3rd or 4th years after introduction, establishment via rosette taken from the arnica collection or early produced can be taken into account.

The highest yield of flower heads was provided by 3- and 4-year-old plants under field cultivation [29, 35, 36, 41]. In the case of plants introduced as rosettes and by sowing, the values of all the parameters studied increased to the 4th year of cultivation and decreased in the next years of observation. Despite this fact, the cultivation should by continued, since the total area of flower heads produced by genets in 2011 and 2012 years introduced through the first mode was comparable to the values noted in 2008 (the second year of cultivation). However, the total area of flower heads of the 5- and 6-year-old plants introduced by sowing was only about 30% lower in relation to the 3- or 4-year-old plants and over 4 times higher compared to the 2-year-old plants.

After the 4th vegetative season, the cumulative total area of flower heads per genet in plants introduced as rosettes and by sowing was comparable. However, in the last two years of the experiment, the values of the characteristics in plants introduced by sowing were higher in relation to plants introduced as rosettes. Additionally, the observed higher survival of plants established by seeds as compared with the other modes of introduction suggests that sowing is better in the perspective of 5-6-year arnica cultivation.

### 4.4. Genet Disintegration

Our studies indicate that in very favourable field conditions, without inter- and intraspecies competition (common in natural habitats), *A. montana* can produce clumping ramets which are developed from the short rhizomes of the spreading ramets, thereby representing a phalanx growth form [42, 43]. *A. montana* genets have a compact structure, but in the 4th or 5th year of observation (depending on the way of introduction), we noticed necromass accumulation and lack of production of new ramets in the central part of the genets. In our opinion, this is the first symptom of the beginning of genet division. The disintegration of genets depended on plant age and is the beginning of vegetative propagation in many clonal plants [44, 45], which had not been described in arnica. After six years of the experiment, we found that the proportion of disintegrated genets depended on the mode of plant propagation and was a consequence of genet senescence. All the genets established as clones were divided. In the case of plants established as rosettes and by sowing, the proportion of disintegrated genets was 84% and 45%, respectively (data not presented).

### 4.5. Arnica Cultivation under the Climatic and Edaphic Conditions

The study of the impact of climate conditions on the traitsstudied over time is difficult and requires many years of observation and therefore is rarely undertaken. Pljevljakušić et al. [20] have presented climatic conditions for three years (2008–2010) of *A. montana* cultivation in Serbia, but did not describe the relation between the climatic factors and the vegetative and reproductive traits. In our experiment, the precipitation patterns of the last two experimental years were different from the previous years. In 2011, the sum of precipitation was over 40% lower in relation to 2009 and 2010, and in 2012, it was over 30% lower, respectively (Figure 1). Therefore, the drastic decrease in the values of some studied traits in the case of plants introduced as clone seedlings in relation to others modes in the last two years of the experiment may have been caused by the lowest sum of precipitation, especially in the spring months. However, it should be underlined that the plants whose traits were compared differed in age, which had an impact on the beginning of genet defragmentation and values of the traits studied over time.

In nature, *A. montana* grows mainly in siliceous mountains, but it also occurs at lower altitudes [3–7, 10, 11]. This species prefers acid and poor soil conditions and is usually dependent on their symbiotic partner [12, 38]. In Poland, arnica grows in lowland areas on acid forest soils (pH 4-4.5) and in mountain areas on acidophilus subalpine grassland soils of pH 4.8-5.5 [38]. In our opinion, arnica plants introduced for cultivation should be cultivated in soil conditions similar to those prevailing in nature. However, in the case of our studies, the plantation was established on grey-brown podsolic soil with acidic reaction and the granulometric composition of heavy loamy sand. The topsoil was characterized by an average content of organic matter, high phosphorus, average potassium, and very low magnesium. The physicochemical soil properties are comparable to natural arnica habitats [7, 9, 38]. Given the growth and *A. montana* reproduction, it can be affirmed that the cultivated plants have favourable edaphic conditions.

## 5. Conclusion

1. The particular ways of *A. montana* plant propagation and introduction presented in this work determined all the intrinsic species traits studied and plant survival.
2. The presented results in relation to another European data indicated that climatic conditions and soil conditions are favourable for arnica cultivation in eastern Poland.
3. Practical implications from the data presented here include the possibility of using the studied ways of *A. montana* propagation and introduction in plant cultivation, which decreases pressure to natural populations in other European countries. We suggest using the three different modes of arnica propagation and introduction in the short- and long-term perspective of arnica cultivation, which can give a possibility of better adjustment of raw material production.
4. From the perspective of multiyear observation of *A. montana* in the field conditions, we can pay attention to genet disintegration, which is an important aspect of the biology of the species. The genet division is the beginning of plant vegetative reproduction, and is determined as vegetative and reproductive traits. This phenomenon is very important for the demography, dynamics, and genetic variation of a natural population and should be studied in the future.

## Figures and Tables

**Table 1:** The effects of three different ways of introduction into cultivation on the studied traits of *A. montana* genets (ANOVA, Kruskall-Wallis test). The *F* and *H* values are shown. The levels of significance are indicated by an asterisk (*0.01 < *P* ≤ 0.05; **0.001 < *P* ≤ 0.01; ***P ≤ 0.001). Explanation: see Section 2.3.

	2007	2008	2009	2010	2011	2012
TNR^*F*^	40.24***	50.48***	34.17***	19.01***	178.90**	256.02**
NFR^*F*^	—	175.07**	15.27***	47.04***	187.19**	382.72**
HS^*H*^	—	67.39***	67.39***	67.39***	67.39***	67.39***
NH^*H*^	—	21.05***	43.09**	46.52***	64.98***	66.59***
NHFS^*F*^	—	15.59***	3.64*	1335.22***	24.06***	43.82***
AH^*H*^	—	21.05***	43.09***	46.52***	64.98***	66.59***
CAH^*H*^	—	62.44***	27.54***	59.88***	65.40***	72.14***
CNH^*F*^	—	389.97**	132.75**	18.45***	179.39**	573.11**
Survival^*H*^	6.5	5.61	7.68*	14.89***	13.77***	15.32***
